# Assessment of respiratory system compliance with electrical impedance tomography using a positive end-expiratory pressure wave maneuver during pressure support ventilation: a pilot clinical study

**DOI:** 10.1186/s13054-014-0679-6

**Published:** 2014-12-10

**Authors:** Tobias H Becher, Simon Bui, Günther Zick, Daniel Bläser, Dirk Schädler, Norbert Weiler, Inéz Frerichs

**Affiliations:** Department of Anesthesiology and Intensive Care Medicine, University Medical Center Schleswig-Holstein, Campus Kiel, Arnold-Heller-Strasse 3, Haus 12, 24105 Kiel, Germany

## Abstract

**Introduction:**

Assessment of respiratory system compliance (C_rs_) can be used for individual optimization of positive end-expiratory pressure (PEEP). However, in patients with spontaneous breathing activity, the conventional methods for C_rs_ measurement are inaccurate because of the variable muscular pressure of the patient. We hypothesized that a PEEP wave maneuver, analyzed with electrical impedance tomography (EIT), might be suitable for global and regional assessment of C_rs_ during assisted spontaneous breathing.

**Methods:**

After approval of the local ethics committee, we performed a pilot clinical study in 18 mechanically ventilated patients (61 ± 16 years (mean ± standard deviation)) who were suitable for weaning with pressure support ventilation (PSV). For the PEEP wave, PEEP was elevated by 1 cmH_2_O after every fifth breath during PSV. This was repeated five times, until a total PEEP increase of 5 cmH_2_O was reached. Subsequently, PEEP was reduced in steps of 1 cmH_2_O in the same manner until the original PEEP level was reached. C_rs_ was calculated using EIT from the global, ventral and dorsal lung regions of interest. For reference measurements, all patients were also examined during controlled mechanical ventilation (CMV) with a low-flow pressure-volume maneuver. Global and regional C_rs_(low-flow) was calculated as the slope of the pressure-volume loop between the pressure that corresponded to the selected PEEP and PEEP +5 cmH_2_O. For additional reference, C_rs_ during CMV (C_rs_(CMV)) was calculated as expired tidal volume divided by the difference between airway plateau pressure and PEEP.

**Results:**

Respiratory system compliance calculated from the PEEP wave (C_rs_(PEEP wave)) correlated closely with both reference measurements (*r* = 0.79 for C_rs_(low-flow) and *r* = 0.71 for C_rs_(CMV)). No significant difference was observed between the mean C_rs_(PEEP wave) and the mean C_rs_(low-flow). However, a significant bias of +17.1 ml/cmH_2_O was observed between C_rs_(PEEP wave) and C_rs_(CMV).

**Conclusion:**

Analyzing a PEEP wave maneuver with EIT allows calculation of global and regional C_rs_ during assisted spontaneous breathing. In mechanically ventilated patients with spontaneous breathing activity, this method might be used for assessment of the global and regional mechanical properties of the respiratory system.

**Electronic supplementary material:**

The online version of this article (doi:10.1186/s13054-014-0679-6) contains supplementary material, which is available to authorized users.

## Introduction

Application of positive end-expiratory pressure (PEEP) is an essential part of ventilator therapy for patients with respiratory failure [1-6]. Despite intensive research in the field, the optimal strategy for individual adjustment of PEEP is still under debate. One strategy that may lead to a PEEP setting that maintains lung recruitment without excessive overdistension is to set PEEP 2 cmH_2_O above the lower inflection point of a static or quasi-static (“low-flow”) pressure-volume loop [7]. This results in ventilation in the area of the pressure-volume loop that is associated with the highest respiratory system compliance (C_rs_). This approach was part of the lung-protective ventilation strategy successfully applied in two randomized and controlled trials [2,3].

However, the global lower inflection point does not accurately reflect the regional mechanical properties of the respiratory system in acute respiratory distress syndrome (ARDS) [8]. Therefore, to prevent regional alveolar collapse and overdistension, a regional assessment of respiratory system mechanics should be applied. Electrical impedance tomography (EIT) is a non-invasive, radiation-free technique that is suitable for regional measurement of C_rs_ [9-12]. Using EIT, regional inflection points can be identified that may be significantly different in the ventral and dorsal parts of the lung [13]. The regional changes in C_rs_, determined by EIT, are closely correlated to overdistension and tidal recruitment [9,10]. This might be used for the selection of ventilator settings that minimize these deleterious phenomena and are associated with better outcomes [14].

A valid measurement of C_rs_ is a prerequisite for the application of any method that is based on global or regional assessment of respiratory system mechanics. In patients with spontaneous breathing activity, the calculation of C_rs_ as the ratio between expired tidal volume (V_Te_) and the inspiratory driving pressure (∆P) becomes inaccurate. Performing a low-flow loop is not feasible in patients when respiratory muscle activity is present. Thus, most conventional C_rs_-based methods for PEEP optimization become invalid in the presence of spontaneous breathing activity.

Iotti *et al*. proposed a method for determination of C_rs_ during pressure support ventilation (PSV) using a least squares fit approach. However, their approach requires the pressure support to be set to a very high level, rendering the patient’s muscular effort negligible [15]. This could be counterproductive for the weaning process and may not be suitable in patients with high respiratory drive.

A PEEP wave maneuver is an alternative method for the determination of the pressure-volume relationship of the respiratory system. Using this method, C_rs_ is determined by measuring the PEEP-induced change in end-expiratory lung volume (∆EELV). Originally, this was done by measuring the difference between inspired tidal volume and V_Te_ before and after a stepwise increments in PEEP [16,17]. Because EIT is able to determine ∆EELV regionally [18,19], analyzing the PEEP wave with EIT could provide insight into the regional pressure-volume relationship of the respiratory system. Theoretically, this approach may be suitable for determination of regional C_rs_ during assisted mechanical ventilation.

We hypothesized that analyzing a PEEP wave maneuver with EIT could be used for global and regional determination of C_rs_ in patients with spontaneous breathing activity.

## Materials and methods

### Patients

We performed a pilot clinical study in the surgical intensive care units (ICUs) of the University Medical Center Schleswig-Holstein, Campus Kiel. Ethical approval was obtained from the ethics committee of the Christian Albrechts University in Kiel, Germany. The study was conducted in compliance with the Helsinki declaration. We included 18 patients (5 women and 13 men; age 61 ± 16 years (mean ± standard deviation)) who were endotracheally intubated and mechanically ventilated in the ICU. All patients were already being ventilated with PSV or clinically suitable for ventilation with PSV at the time of inclusion. Exclusion criteria were age <18 years, pregnancy, open-chest injury, unstable spinal injury, hemodynamic instability and severe chronic obstructive pulmonary disease. Written informed consent was obtained from all patients or their legal representatives. Detailed patient characteristics are given in Table 1. Additional information on ventilator settings and patient work of breathing is given in Table 2.

**Table 1:** **Patients’ characteristics**

**Patient**	**Height (cm)**	**Airway (ETT/TT; mm ID)**	**MV duration (days)**	**Diagnosis**
1	180	ETT; 8.0	12	ARDS (moderate)
2	168	ETT; 7.5	4	Sepsis
3	175	TT; 9.0	22	ARDS (mild)
4	158	ETT; 7.0	9	ARDS (moderate)
5	185	ETT; 8.0	8	ARDS (moderate)
6	170	ETT; 8.5	4	ARDS (moderate)
7	170	ETT; 8.0	6	ARDS (moderate)
8	166	ETT; 8.0	6	ARDS (moderate)
9	160	ETT; 7.5	1	ARDS (mild)
10	164	ETT; 8.5	1	Postop.
11	171	ETT; 7.5	7	Sepsis
12	179	TT; 9.0	24	ARDS (mild)
13	176	ETT; 8.5	1	Postop.
14	166	ETT; 7.5	5	Cardiac failure
15	163	TT; 9.0	15	ARDS (moderate)
16	178	ETT; 8.5	1	Postop.
17	182	TT; 9.0	5	ARDS (moderate)
18	170	ETT; 8.5	7	ARDS (mild)
Mean	171	–	8	–
SD	8	–	7	–

Acute respiratory distress syndrome (ARDS) severity was assessed according to the Berlin definition [20]. ETT: Endotracheal tube; TT: Tracheostomy tube; ID: Inner diameter; MV duration: Days of mechanical ventilation prior to the study, with the day of study procedure included; Postop.: Patients without pulmonary pathology examined after scheduled major surgery.

**Table 2:** **Ventilator settings**

**Patient**	**RR**_**CMV**_ **(1/min)**	**V**_**T,CMV **_ **(ml)**	**P**_**plat,CMV **_ **(cmH**_**2**_ **O)**	**PEEP ** **(cmH**_**2**_ **O)**	**RR**_**PSV **_ **(1/min)**	**V**_**T,PSV**_ **(ml)**	**PS ** **(cmH**_**2**_ **O)**	**WOB ** **(J/L)**
1	15	550	23	10	14	510	10	0.35
2	16	520	24	10	16	530	12	0.96
3	35	300	35	10	15	440	10	1.23
4	27	270	36	15	18	400	15	0.91
5	14	540	20	10	13	660	10	0.46
6	10	710	30	12	16	670	13	0.98
7	18	510	24	15	16	540	10	0.93
8	13	500	29	15	17	460	9	0.48
9	13	510	23	11	18	510	8	1.02
10	12	520	12	5	17	690	6	0.19
11	15	550	16	5	16	820	7	0.84
12	20	490	19	7	14	500	12	0.58
13	22	540	25	15	15	750	10	0.53
14	15	480	22	8	17	600	12	1.11
15	15	390	33	13	17	620	12	0.84
16	10	640	21	10	14	400	9	0.48
17	18	420	20	12	14	920	8	0.42
18	13	600	19	8	16	480	10	0.59
Mean	17	501	24	11	16	585	10	0.72
SD	6	104	6	3	1	131	2	0.29

CMV: Controlled mechanical ventilation; RR_CMV_: Respiratory rate under CMV; V_T,CMV_: Tidal volume under CMV; P_plat,CMV_: Plateau pressure under CMV; PEEP: Clinically selected positive end-expiratory pressure; RR_PSV_: Respiratory rate under pressure support ventilation (before the start of the PEEP wave maneuver); V_T,PSV_: Mean tidal volume during pressure support ventilation before the start of the PEEP wave maneuver; PS: Pressure support during PSV; WOB: Patient work of breathing during undisturbed PSV.

### Ventilator procedure and data acquisition

All patients were ventilated with Evita XL ventilators (Dräger Medical, Lübeck, Germany). The examinations were carried out with the patients in the supine or semirecumbent position. The level of pressure support and the initial PEEP were selected according to clinical criteria by the physician in charge, with the aim of achieving a respiratory rate <30/min and a tidal volume in the range of 5 to 10 ml/kg predicted body weight. Airflow and airway pressure (P_aw_) were recorded from the ventilator at a sampling rate of 125 Hz. Volume was calculated by mathematical integration of the flow signal. Additionally, airflow, P_aw_ and esophageal pressure (P_es_) were recorded with the BiCore 2 measurement device (CareFusion, Yorba Linda, CA, USA) at a sampling rate of 100 Hz. Correct positioning of the esophageal probe was confirmed by visual analysis of cardiac oscillations and by performing an end-expiratory occlusion test as described by Baydur *et al*. [21].

EIT data were acquired using the Goe-MF II device (CareFusion). Sixteen self-adhesive electrodes (Blue Sensor L-00-S; Ambu, Ballerup, Denmark) were placed around the chest circumference in one transverse plane lying approximately at the level of the fifth intercostal space. EIT images were obtained at a scan rate of 25 Hz.

### Controlled mechanical ventilation

To obtain reference values for C_rs_, all patients were deeply sedated to a score of −5 on the Richmond Agitation and Sedation Scale (RASS) [22]. If spontaneous breathing activity (as evidenced by careful observation of the flow, P_aw_ and P_es_ curves) persisted at a RASS score of −5, patients were additionally paralyzed with rocuronium bromide in order to temporarily interrupt all spontaneous breathing activity. At the same time, the ventilator mode was changed from PSV to CMV. During CMV, patients were ventilated with a V_Te_ of 8 ± 2 ml/kg predicted body weight. Inspiratory flow was adjusted to reach an end-inspiratory pause (T_plat_) of 0.8 ± 0.3 seconds. Airway plateau pressure (P_plat_) was measured at the end of T_plat_. After a short phase of CMV, a low-flow pressure-volume maneuver was performed by the ventilator with a constant gas flow of 4 L/min, starting at 0 P_aw_ up to a maximum volume of 2 L or a maximum P_aw_ of 35 cmH_2_O. After this maneuver, patients were ventilated with CMV until the effects of the applied sedatives and (if applicable) neuromuscular blocking agents had subsided. After the return of sufficient spontaneous breathing activity, the ventilator mode was changed back to PSV with the previous settings. The PEEP level remained unchanged when the ventilator modes were switched from PSV to CMV and vice versa.

### PEEP wave maneuver

**Figure 1:**
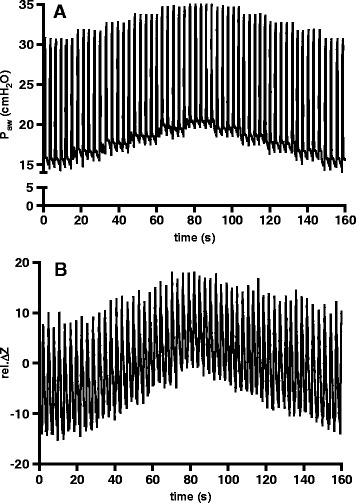
**Airway pressure and electrical impedance tomography waveforms acquired during the positive end-expiratory pressure wave maneuver in one of the studied patients. (A)** Airway pressure (P_aw_) during the maneuver. Positive end-expiratory pressure was elevated by 1 cmH_2_O after every fifth breath while the pressure support level remained constant. **(B)** Time course of global relative impedance changes (rel.∆Z) during the maneuver.

The PEEP wave maneuver was executed during PSV in a phase of stable spontaneous breathing. The flow trigger was set to 2 L/min, and the PS termination criterion was adjusted to 25% of peak inspiratory flow. The dosing of sedatives was adjusted to achieve a RASS score of −3 to −4. For the maneuver, the PEEP level was elevated by 1 cmH_2_O after five consecutive PSV breaths at the initial PEEP level. After another five breaths, PEEP was elevated by another 1 cmH_2_O. This procedure was repeated five times until a total PEEP increase of 5 cmH_2_O compared to the initial value was achieved. Afterward, PEEP was lowered in increments of 1 cmH_2_O in the same way (Figure 1).

### Sequence of measurements

The sequence of measurements was defined randomly. In nine patients, we performed the PEEP wave before the reference measurements; in the other nine patients, the reference measurements were performed before the PEEP wave.

### Electrical impedance tomography image generation and analysis

**Figure 2:**
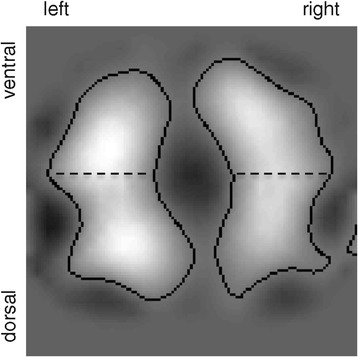
**Example of the functional region of interest definition.** The region of interest was divided into its ventral and dorsal parts along the dashed line.

Cross-sectional images were calculated from EIT data using a normalized difference reconstruction algorithm based on the Graz consensus reconstruction algorithm for EIT [23]. To minimize artifacts caused by cardiac oscillations, a low-pass filter with a cutoff frequency set at 50 Hz, which in all cases was below the patients’ heart rates, was employed. A functional region of interest (ROI) was selected individually for every patient using the regression slope method [24] on the EIT data that had been recorded during the PEEP wave. For regional analysis, the ROI was divided into a ventral part and a dorsal part along a horizontal line. This line was placed exactly in the middle of the vertical lung region dimension (Figure 2) by dividing the total number of horizontal rows of EIT data in the ROI by 2. In cases of an uneven number of rows, the remaining row was added to the dorsal part of the ROI. Once the functional lung ROI had been defined for an individual patient using the EIT images generated during the PEEP wave maneuver, the same ROI was applied for all further analyses of the PEEP wave and the reference measurements in CMV.

### Calculation of respiratory system compliance from positive end-expiratory pressure wave

**Figure 3:**
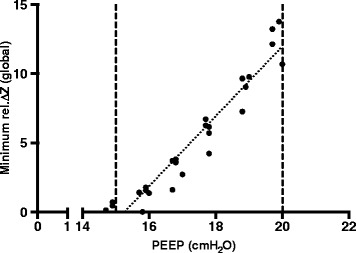
**Calculation of the slope of the change in global impedance minima per 1 cmH**_**2**_ **O during the positive end-expiratory pressure wave in one of the studied patients.** Each data point represents the global impedance minimum of one breath at the corresponding positive end-expiratory pressure value. The dotted line shows the result of the linear regression fit (“best-fit line”). The dashed lines indicate the investigated pressure range. rel.∆Z: Relative impedance change.

To obtain a calibration factor between tidal impedance change (∆Z) and V_Te_, the mean V_Te_ during 4 breaths at the initial PEEP level was divided by the mean global tidal ∆Z during the same breaths. To obtain the relationship between PEEP and ∆Z, the slope of the mean change in global impedance minima per cmH_2_O during the PEEP wave was calculated using a least-squares approximation (Figure 3). The obtained slope was then multiplied with the aforementioned calibration factor to calculate the global value of C_rs_(PEEP wave) in ml/cmH_2_O.

For regional analysis of C_rs_ during the PEEP wave, the slopes of the mean change in impedance minima were calculated separately for the ventral and dorsal lung ROIs. The respective values were then multiplied by the global calibration factor to obtain the regional values of C_rs_(PEEP wave)_ventral_ and C_rs_(PEEP wave)_dorsal_. Because the ventral and dorsal parts of the functional lung ROI may contain a different number of image pixels, normalized regional per-pixel values of C_rs_(PEEP wave) were calculated by dividing C_rs_(PEEP wave)_ventral_ and C_rs_(PEEP wave)_dorsal_ by the total number of image pixels in the respective parts of the ROI.

### Calculation of reference values for respiratory system compliance

**Figure 4:**
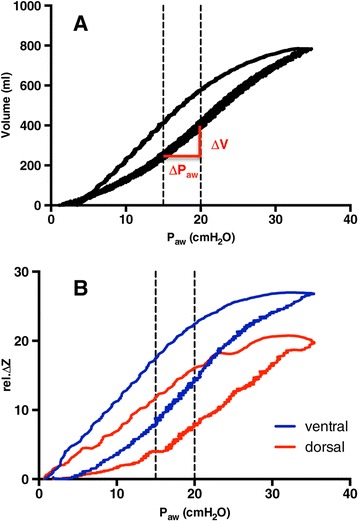
**Low-flow pressure-volume loop. (A)** Example of a global low-flow pressure-volume loop in one of the studied patients. The slope of global volume change (∆V) divided by the corresponding change in airway pressure (∆P_aw_) was calculated in the pressure range that was examined with the positive end-expiratory pressure (PEEP) wave, providing a reference value for global respiratory system compliance (C_rs_(low-flow)). The dashed lines indicate the pressure range investigated during the PEEP wave maneuver. **(B)** Regional pressure-impedance (rel.∆Z) loops obtained by electrical impedance tomography during the same maneuver. The slopes of the curves obtained in the ventral and dorsal lung regions were multiplied by C_rs_(low-flow) to yield the reference values of C_rs_(low-flow)_ventral_ and C_rs_(low-flow)_dorsal_, respectively. The dotted lines indicate the investigated pressure range (PEEP − PEEP +5).

To obtain a reference value for quasi-static C_rs_, we calculated the slope of the low-flow pressure-volume loop between the P_aw_ values spanning the PEEP settings during the PEEP wave maneuver. For example, if the PEEP wave had started at a PEEP of 15 cmH_2_O in an individual patient, the slope of the low-flow pressure-volume loop was calculated between P_aw_ of 15 cmH_2_O and 20 cmH_2_O to obtain the reference value of C_rs_(low-flow) (Figure 4A).

To obtain reference values of ventral and dorsal C_rs_, the slopes of the mean changes in ventral, dorsal and global impedance minima per 1 cmH_2_O P_aw_ were calculated from the same sections of the low-flow loops (Figure 4B). The ventral and dorsal fractions of C_rs_ were then calculated by dividing the respective slope values by the global slope. C_rs_(low-flow)_ventral_ and C_rs_(low-flow)_dorsal_ were then obtained by multiplying the fractional ventral and dorsal slopes by the global C_rs_(low-flow). The regional per-pixel values of C_rs_(low-flow) were calculated by dividing the ventral and dorsal values by the total number of image pixels in the respective parts of the ROI.

For additional reference, C_rs_ during controlled mechanical ventilation (C_rs_(CMV)) was calculated as V_Te_ divided by the difference between P_plat_ and PEEP:

$$ {\mathrm{C}}_{\mathrm{rs}}\left(\mathrm{C}\mathrm{M}\mathrm{V}\right) = {\mathrm{V}}_{\mathrm{Te}}/\left({\mathrm{P}}_{\mathrm{plat}} - \mathrm{PEEP}\right) $$

### Statistical analysis

All data were tested for normal distribution using the D’Agostino-Pearson omnibus normality test with a threshold α = 0.05. The interpatient correlations between C_rs_ assessed with the PEEP wave maneuver and the reference values for C_rs_ were calculated with the Pearson correlation for normally distributed data and with the Spearman correlation for non-normally distributed data. Additionally, all data were compared with the Bland-Altman analysis. The differences between the mean results of the methods were tested for statistical significance using a paired *t*-test.

## Results

All analyzed data passed the normality test. Thus, the Pearson correlation could be calculated for all data sets analyzed.

The mean values and standard deviations of C_rs_, assessed with the different methods, are presented in Table 3. Individual C_rs_ values are provided in Table S1 and Table S2 in Additional file 1. An overview of all correlations, bias and limits of agreement is given in Table S3 in Additional file 1.

**Table 3:** **Means and standard deviations of the analyzed values of respiratory system compliance**

**Value**	**Mean ± SD ** **(ml/cmH**_**2**_ **O)**
C_rs_(PEEP wave)	59.8 ± 20.8
C_rs_(PEEP wave)_ventral_	40.9 ± 15.9
C_rs_(PEEP wave)_dorsal_	18.8 ± 9.3^a^
C_rs_(PEEP wave) per pixel	0.20 ± 0.09
C_rs_(PEEP wave)_ventral_ per pixel	0.26 ± 0.13
C_rs_(PEEP wave)_dorsal_ per pixel	0.14 ± 0.07^b^
C_rs_(low-flow)	53.0 ± 22.4
C_rs_(low-flow)_ventral_	36.2 ± 16.5
C_rs_(low-flow)_dorsal_	16.8 ± 8.8^a^
C_rs_(low-flow) per pixel	0.18 ± 0.07
C_rs_(low-flow)_ventral_ per pixel	0.23 ± 0.11
C_rs_(low-flow)_dorsal_ per pixel	0.12 ± 0.06^a^
C_rs_(CMV)	47.1 ± 21.6

^a^Significantly different from the corresponding value in the ventral region (*P* <0.0001). ^b^Significantly different from the corresponding value in the ventral region (*P* = 0.0002). Per-pixel values were obtained by dividing the ventral and dorsal values of C_rs_ by the total number of image pixels in the respective regions of interest. C_rs_(PEEP wave): Respiratory system compliance obtained by performing the positive end-expiratory pressure (PEEP) wave maneuver during pressure support ventilation; C_rs_(low-flow): Quasi-static C_rs_ obtained with the low-flow loop during controlled mechanical ventilation; C_rs_(CMV): C_rs_ calculated by dividing expiratory tidal volume by the difference between plateau airway pressure and PEEP during controlled mechanical ventilation.

### Global respiratory system compliance

Comparing C_rs_(PEEP wave) to the reference values, we found a highly significant correlation with the reference value C_rs_(low-flow) (*r* = 0.80; *P* <0.0001; C_rs_(PEEP wave) = 0.73 × C_rs_(low-flow) +21). There was a clear trend (*P* = 0.06) toward a higher mean C_rs_(PEEP wave) of 6.8 ml/cmH_2_O in comparison to C_rs_(low-flow).

In comparison to the second reference value, C_rs_(CMV), we found a similar degree of correlation that was also highly significant (*r* = 0.71; *P* = 0.001; C_rs_(PEEP wave = 0.91 × C_rs_(CMV) +21). The mean C_rs_(PEEP wave) was +17.1 ml/cmH_2_O higher than the mean C_rs_(CMV). This difference was statistically significant (*P* = 0.0002).

**Figure 5:**
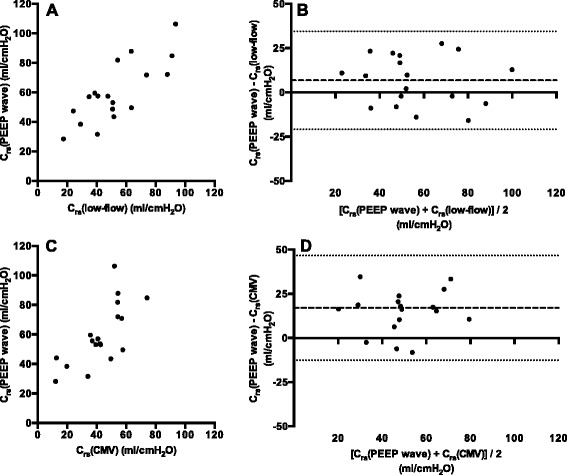
**Comparison of global values for respiratory system compliance. (A)** Correlation between the global respiratory system compliance (C_rs_), determined with electrical impedance tomography during assisted spontaneous breathing with the (PEEP) wave maneuver (C_rs_(PEEP wave)), and quasi-static C_rs_, determined in the passive patient with a low-flow pressure-volume loop (C_rs_(low-flow). **(B)** Bland-Altman method comparison between C_rs_(PEEP wave) and C_rs_(low-flow). The dashed line indicates the bias (+6.8 ml/cmH_2_O), and the dotted lines indicate the 95% limits of agreement (−20.8 to +34.5 ml/cmH_2_O). **(C)** Correlation between C_rs_(PEEP wave) and C_rs_, determined during volume-controlled ventilation (C_rs_(CMV)). **(D)** Bland-Altman method comparison between C_rs_(PEEP wave) and C_rs_(CMV). The dashed line indicates the bias (+17.1 ml/cmH_2_O), and the dotted lines indicate the 95% limits of agreement (−13 to +47 ml/cmH_2_O).

The correlation and the Bland-Altman methods comparison of C_rs_(PEEP wave) with both reference values are shown in Figure 5.

### Regional respiratory system compliance

**Figure 6:**
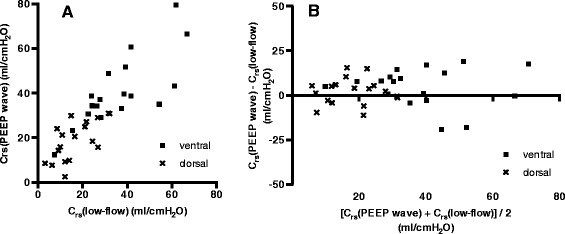
**Comparison of regional values for respiratory system compliance. (A)** Correlation between regional respiratory system compliance (C_rs_), determined using electrical impedance tomography (EIT) during assisted spontaneous breathing with the positive end-expiratory pressure (PEEP) wave maneuver (C_rs_(PEEP wave) and quasi-static regional C_rs_, determined in the passive patient with EIT during a low-flow pressure-volume loop (C_rs_(low-flow)). **(B)** Bland-Altman method comparison between regional C_rs_(PEEP wave) and regional C_rs_(low-flow).

The ventral C_rs_(PEEP wave) was significantly correlated to the ventral C_rs_(low-flow) (*r* = 0.77; *P* = 0.0002). Similar to the global results, there was a trend toward a higher value of ventral C_rs_(PEEP wave) in comparison with ventral C_rs_(low-flow) that did not reach statistical significance. The correlation of the dorsal C_rs_(PEEP wave) with the dorsal C_rs_(low-flow) was weaker than the correlation in the ventral ROIs, but still highly significant (*r* = 0.65; *P* = 0.003). Again, there was a trend toward a higher value of C_rs_(PEEP wave) in comparison to C_rs_(low-flow) that did not reach statistical significance (*P* = 0.24). The regional correlations and Bland-Altman comparisons are shown in Figure 6.

**Figure 7:**
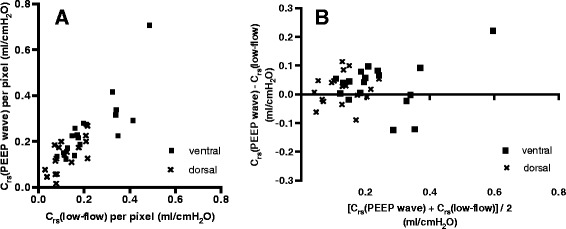
**Comparison of regional per-pixel values for respiratory system compliance. (A)** Correlation between the regional respiratory system compliance (C_rs_), determined with electrical impedance tomography during assisted spontaneous breathing with the positive end-expiratory pressure (PEEP) wave maneuver (C_rs_(PEEP wave)) and quasi-static regional C_rs_, determined in the passive patient with a low-flow pressure-volume loop (C_rs_(low-flow)), after normalizing the regional values to the number of pixels in the ventral and dorsal regions of interest for every patient. **(B)** Bland-Altman method comparison between the regional values, normalized to the number of pixels in the respective regions.

Both the ventral C_rs_(low-flow) and C_rs_(PEEP-wave) values were significantly higher than the corresponding dorsal values (*P* <0.0001). When comparing the normalized per-pixel values, the ventral C_rs_(low-flow) and C_rs_(PEEP wave) values were still significantly higher than their dorsal equivalents (*P* = 0.0002 and *P* <0.0001, respectively). The correlations of the per-pixel C_rs_ values and the Bland-Altman comparisons are depicted in Figure 7.

## Discussion

We performed a pilot clinical study to determine global and regional C_rs_ by performing a PEEP wave maneuver during PSV that was subsequently analyzed with EIT. The calculated values were compared to reference values that had been determined with a low-flow pressure-volume maneuver and during volume-controlled ventilation with T_plat_. We found highly significant correlations between C_rs_(PEEP wave) and the values obtained using the reference methods. However, there was a trend toward a higher mean C_rs_(PEEP wave) compared to the reference C_rs_ values. This difference did not reach statistical significance when we compared C_rs_(PEEP wave) to the quasi-static C_rs_(low-flow), which may have been due to our relatively small sample size; however, it was highly significant when we compared C_rs_(PEEP wave) to C_rs_(CMV).

### Measurement bias

There are several possible explanations for the higher mean value of C_rs_(PEEP wave) compared to the reference values. First, owing to the long equilibration time at every PEEP level, assessment of C_rs_ during a PEEP wave maneuver yields an almost static value. Because the reference value C_rs_(CMV) was calculated by dividing V_Te_ by the difference between P_plat_ measured after an end-inspiratory occlusion of only 0.8 ± 0.3 seconds, it was likely to be lower than the “static” C_rs_(PEEP wave) due to the viscoelastic properties of the patient’s respiratory system [25]. Moreover, especially in patients with ARDS, C_rs_ is usually nonlinear and tends to be lower at higher levels of airway pressure [26]. Because the values of P_plat_ were obviously much higher than the pressure range we investigated with our PEEP wave maneuver, this effect is likely to have contributed to the difference between C_rs_(PEEP wave) and C_rs_(CMV).

Another explanation for the observed differences could be the fact that spontaneous breathing may lead to recruitment of lung tissue and to alterations of chest wall mechanics [27]. This could have contributed to the higher C_rs_ we found with the PEEP wave during assisted spontaneous breathing. Additionally, the PEEP wave itself could also cause recruitment by an increase in mean and peak airway pressures during the maneuver.

The observed global increase in impedance may in part have been caused by a displacement of blood out of the thorax resulting from the change in mean airway pressure. Such an effect would lead to an increase in global impedance within the lung ROI that cannot easily be distinguished from an increase caused by rising EELV. However, because the displacement of blood is accompanied by a simultaneous increase in aerated lung volume, the absolute change in impedance due to changes in blood volume may be negligible as compared to the concomitant changes in aerated lung volume [28].

### Accuracy of the proposed method

Although we found a good general correlation between C_rs_(PEEP wave) and the reference values, considerable differences were observed between these values in some patients. Also, the overall 95% limits of agreement were relatively broad when we compared C_rs_(PEEP wave) to the reference measurements. This may in part be caused by alterations in respiratory system mechanics induced by spontaneous breathing. Additionally, one must bear in mind that the PEEP wave maneuver during PSV is based on the assumption that that patient’s respiratory muscles reach a more or less relaxed state at the end of expiration. In patients who exhibit an irregular breathing pattern or who use their expiratory muscles actively to counterbalance the effects of PEEP, the measurement of C_rs_ with a PEEP wave may become inaccurate. For our study, the PEEP wave maneuver was executed during a relatively deep level of sedation (RASS −3 to −4) in order to achieve a state of relaxed spontaneous breathing. However, because there were no predefined exclusion criteria related to the patient’s breathing pattern, expiratory muscle activity or irregular breathing patterns may still have been present in some of our patients.

For broad application of the proposed method during PSV, it might be necessary to define exclusion criteria based on the patient’s breathing pattern in order to avoid faulty measurements. It should then be possible to test the proposed method in patients under lighter sedation.

### Regional analysis

In our regional analysis, we found a significantly higher C_rs_(PEEP wave) as well as C_rs_(low flow) in the ventral ROI when compared to the dorsal ROI. Because the ROIs were divided along a horizontal line in the middle of their vertical dimensions, this may have resulted in a different number of pixels in the ventral and dorsal ROIs. In fact, there were slightly more image pixels in the ventral part of the ROI (166 ± 44 (mean ± SD) ventral vs. 143 ± 23 dorsal; *P* = 0.02). However, after normalizing the regional C_rs_ to the number of pixels in the respective parts of the ROI, there was still a significantly higher C_rs_ in the ventral part, when assessed with the PEEP wave and with the low-flow loop. Therefore, the differences between ventral and dorsal C_rs_ cannot be explained by the number of pixels alone.

A likely explanation for the higher ventral values of C_rs_(PEEP wave) and C_rs_(low-flow) is the different shape of the ventral and dorsal pressure-volume loop. As can be seen in the example in Figure 4, the dorsal slope was frequently smaller than the ventral one in the analyzed pressure range (PEEP − PEEP +5 cmH_2_O). It is likely that with a higher initial PEEP (for example, a PEEP >20 cmH_2_O in the patient in Figure 4), we would have found similar ventral and dorsal values of C_rs_. With an even higher PEEP (for example, a PEEP >25 cmH_2_O in the patient in Figure 4), we would have found a lower ventral C_rs_ because of regional overdistension. One can speculate that the “best PEEP” setting would be a PEEP that leads to maximum dorsal C_rs_ while avoiding a significant decrease in ventral C_rs_ due to overdistension.

### Clinical relevance and feasibility

The PEEP wave is a simple maneuver that can be executed repeatedly without any negative effects for the patient. In contrast, the low-flow loop requires temporary interruption of spontaneous breathing activity. In patients who are in the early weaning phase from mechanical ventilation, the administration of sedatives or even neuromuscular blocking agents is not desirable, because it may lead to an unnecessary prolongation of the weaning process. In these cases, a repeated PEEP wave maneuver—for example, before and after a decremental PEEP trial—may help in finding an individual PEEP setting. For example, the PEEP setting might be adjusted to optimize C_rs_ in the dorsal ROI in order to avoid opening and closing of alveoli. This approach would be similar to the one that leads to improved gas exchange and lung mechanics and reduced histologic evidence of lung injury in an animal model of ARDS during CMV [14], but it would also be feasible in patients with spontaneous breathing activity.

Performing the PEEP wave maneuver and its analysis “by hand,” as we did in our present study, is a rather laborious and error-prone task. An alternative method could be to perform a single PEEP step of 5 cmH_2_O and to wait for 20 breaths to measure ∆EELV. However, we chose not to carry out a single PEEP step of 5 cmH_2_O, because we assumed that this would have disrupted the patient’s breathing pattern by causing coughing, forced expiration or other undesirable respiratory reflex effects disturbing the measurement of ∆EELV.

In the future, the PEEP wave maneuver could be performed automatically by the ventilator, as it has previously been shown to be possible for CMV in a previous version of the Evita respirator [16]. The recording of a static pressure-volume loop with the super-syringe technique is an example of an even more complicated maneuver that has been greatly simplified by its automatic implementation on many ventilators in the form of a low-flow pressure-volume loop. Implementation of the PEEP wave in ventilator software as a measurement maneuver during PSV, analogous to the low-flow inflation-deflation maneuver, would make the method suitable for daily clinical use. Similarly, the regional analysis could be done automatically with a modified version of the EIT software.

## Conclusions

We present a method for global and regional assessment of C_rs_ during assisted spontaneous breathing with a PEEP wave maneuver that was analyzed with EIT. In general, the method showed good correlations to the reference values for global and regional C_rs_ recorded during CMV and during a low-flow pressure-volume loop. Performing repeated PEEP wave maneuvers starting from different PEEP levels could be suitable for identifying the PEEP level that leads to optimal dorsal lung recruitment in patients with spontaneous breathing activity.

## Key messages

- A PEEP wave is a short ventilation maneuver with stepwise successive increases and decreases in PEEP by 1 cmH_2_O for a few breaths at each step.
- A PEEP wave maneuver can be used for determination of C_rs_ in mechanically ventilated patients during assisted spontaneous breathing.
- Analyzing the PEEP wave with EIT allows regional assessment of C_rs_ during assisted spontaneous breathing.
- Repeated PEEP wave maneuvers starting from different PEEP levels could be used for identifying the PEEP level that maintains optimal dorsal lung recruitment in patients with spontaneous breathing activity.

## Additional file

Additional file 1:
**Overview of all C**_**rs**_ **values and of all correlations, bias and limits of agreement.** Individual C_rs_ values are provided in Table S1 and Table S2. An overview of all correlations, bias and limits of agreement is given in Table S3. Table S1: Overview of all global C_rs_ values. Table S2: Overview of all regional C_rs_. Table S3: Overview of all correlations, bias and limits of agreement.

## Abbreviations

∆P: Inspiratory driving pressure (P_aw_ − PEEP)
∆Z: Impedance change
ARDS: Acute respiratory distress syndrome
CMV: Controlled mechanical ventilation
C_rs_(CMV): Respiratory system compliance calculated as V_Te_/(P_plat_ − PEEP)
C_rs_(low flow): Respiratory system compliance calculated from the low-flow pressure-volume loop
C_rs_(PEEP wave): Respiratory system compliance calculated from the PEEP wave
C_rs_: Respiratory system compliance
EELV: End-expiratory lung volume
EIT: Electrical impedance tomography
ICU: Intensive care unit
P_aw_: Airway pressure
PEEP: Positive end-expiratory pressure
P_es_: Esophageal pressure
P_plat_: Airway plateau pressure
PSV: Pressure support ventilation
RASS: Richmond Agitation and Sedation Scale
ROI: Region of interest
T_plat_: Duration of end-inspiratory pause
V_Te_: Expired tidal volume
V_Ti_: Inspired tidal volume

## References

[1] Suter PM, Fairley B, Isenberg MD (1975). Optimum end-expiratory airway pressure in patients with acute pulmonary failure. N Engl J Med.

[2] Amato MB, Barbas CS, Medeiros DM, Magaldi RB, Schettino GP, Lorenzi-Filho G, Kairalla RA, Deheinzelin D, Munoz C, Oliveira R, Takagaki TY, Carvalho CR (1998). Effect of a protective-ventilation strategy on mortality in the acute respiratory distress syndrome. N Engl J Med.

[3] Villar J, Kacmarek RM, Pérez-Méndez L, Aguirre-Jaime A (2006). A high positive end-expiratory pressure, low tidal volume ventilatory strategy improves outcome in persistent acute respiratory distress syndrome: a randomized, controlled trial. Crit Care Med.

[4] Gattinoni L, Caironi P, Cressoni M, Chiumello D, Ranieri VM, Quintel M, Russo S, Patroniti N, Cornejo R, Bugedo G (2006). Lung recruitment in patients with the acute respiratory distress syndrome. N Engl J Med.

[5] Briel M, Meade M, Mercat A, Brower RG, Talmor D, Walter SD, Slutsky AS, Pullenayegum E, Zhou Q, Cook D, Brochard L, Richard JC, Lamontagne F, Bhatnagar N, Stewart TE, Guyatt G (2010). Higher vs lower positive end-expiratory pressure in patients with acute lung injury and acute respiratory distress syndrome: systematic review and meta-analysis. JAMA.

[6] The Acute Respiratory Distress Syndrome Network (2000). Ventilation with lower tidal volumes as compared with traditional tidal volumes for acute lung injury and the acute respiratory distress syndrome. N Engl J Med.

[7] Gattinoni L, Pesenti A, Avalli L, Rossi F, Bombino M (1987). Pressure-volume curve of total respiratory system in acute respiratory failure: computed tomographic scan study. Am Rev Respir Dis.

[8] Hickling KG (1998). The pressure-volume curve is greatly modified by recruitment: a mathematical model of ARDS lungs. Am J Respir Crit Care Med.

[9] Costa EL, Borges JB, Melo A, Suarez-Sipmann F, Toufen C, Bohm SH, Amato MB (2009). Bedside estimation of recruitable alveolar collapse and hyperdistension by electrical impedance tomography. Intensive Care Med.

[10] Frerichs I, Dargaville PA, van Genderingen H, Morel DR, Rimensberger PC (2006). Lung volume recruitment after surfactant administration modifies spatial distribution of ventilation. Am J Respir Crit Care Med.

[11] Bikker IG, Leonhardt S, Reis Miranda D, Bakker J, Gommers D (2010). Bedside measurement of changes in lung impedance to monitor alveolar ventilation in dependent and non-dependent parts by electrical impedance tomography during a positive end-expiratory pressure trial in mechanically ventilated intensive care unit patients. Crit Care.

[12] Lowhagen K, Lundin S, Stenqvist O (2010). Regional intratidal gas distribution in acute lung injury and acute respiratory distress syndrome–assessed by electric impedance tomography. Minerva Anestesiol.

[13] van Genderingen HR, van Vught AJ, Jansen JR (2003). Estimation of regional lung volume changes by electrical impedance pressures tomography during a pressure-volume maneuver. Intensive Care Med.

[14] Wolf GK, Gómez-Laberge C, Rettig JS, Vargas SO, Smallwood CD, Prabhu SP, Vitali SH, Zurakowski D, Arnold JH (2013). Mechanical ventilation guided by electrical impedance tomography in experimental acute lung injury. Crit Care Med.

[15] Iotti GA, Braschi A, Brunner JX, Smits T, Olivei M, Palo A, Veronesi R (1995). Respiratory mechanics by least squares fitting in mechanically ventilated patients: applications during paralysis and during pressure support ventilation. Intensive Care Med.

[16] Putensen C, Baum M, Koller W, Putz G (1989). The PEEP wave: an automated technique for bedside determination of the volume/pressure ratio in the lungs of ventilated patients [Article in German]. Anaesthesist.

[17] Putensen C, Baum M, Hörmann C (1993). Selecting ventilator settings according to variables derived from the quasi-static pressure/volume relationship in patients with acute lung injury. Anesth Analg.

[18] Frerichs I, Hahn G, Hellige G (1999). Thoracic electrical impedance tomographic measurements during volume controlled ventilation-effects of tidal volume and positive end-expiratory pressure. IEEE Trans Med Imaging.

[19] Meier T, Luepschen H, Karsten J, Leibecke T, Grossherr M, Gehring H, Leonhardt S (2008). Assessment of regional lung recruitment and derecruitment during a PEEP trial based on electrical impedance tomography. Intensive Care Med.

[20] The ARDS Definition Task Force (2012). Acute respiratory distress syndrome: the Berlin Definition. JAMA.

[21] Baydur A, Behrakis PK, Zin WA, Jaeger M, Milic-Emili J (1982). A simple method for assessing the validity of the esophageal balloon technique. Am Rev Respir Dis.

[22] Sessler CN, Gosnell MS, Grap MJ, Brophy GM, O’Neal PV, Keane KA, Tesoro EP, Elswick RK (2002). The Richmond Agitation-Sedation Scale: validity and reliability in adult intensive care unit patients. Am J Respir Crit Care Med.

[23] Adler A, Arnold JH, Bayford R, Borsic A, Brown B, Dixon P, Faes TJ, Frerichs I, Gagnon H, Gärber Y, Grychtol B, Hahn G, Lionheart WR, Malik A, Patterson RP, Stocks J, Tizzard A, Weiler N, Wolf GK (2009). GREIT: a unified approach to 2D linear EIT reconstruction of lung images. Physiol Meas.

[24] Pulletz S, van Genderingen HR, Schmitz G, Zick G, Schädler D, Scholz J, Weiler N, Frerichs I (2006). Comparison of different methods to define regions of interest for evaluation of regional lung ventilation by EIT. Physiol Meas.

[25] D’Angelo E, Calderini E, Torri G, Robatto FM, Bono D, Milic-Emili J (1989). Respiratory mechanics in anesthetized paralyzed humans: effects of flow, volume, and time. J Appl Physiol.

[26] Stahl CA, Möller K, Schumann S, Kuhlen R, Sydow M, Putensen C, Guttmann J (2006). Dynamic versus static respiratory mechanics in acute lung injury and acute respiratory distress syndrome. Crit Care Med.

[27] Kimball WR, Loring SH, Basta SJ, De Troyer A, Mead J (1985). Effects of paralysis with pancuronium on chest wall statics in awake humans. J Appl Physiol.

[28] Faes TJ, van der Meij HA, de Munck JC, Heethaar RM (1999). The electric resistivity of human tissues (100 Hz-10 MHz): a meta-analysis of review studies. Physiol Meas.

