# Supplementary material for: Assessment of respiratory system compliance with electrical impedance tomography using a positive end-expiratory pressure wave maneuver during pressure support ventilation: a pilot clinical study
Source: Crit Care. 2014 Dec 10;18(6):679. doi: 10.1186/s13054-014-0679-6 (PMC4301036; doi:10.1186/s13054-014-0679-6)
Supplement: Additional file 1: — Overview of all C rs values and of all correlations, bias and limits of agreement. Individual Crs values are provided in Table S1 and Table S2. An overview of all correlations, bias and limits of agreement is given in Table S3. Table S1: Overview of all global Crs values. Table S2: Overview of all regional Crs. Table S3: Overview of all correlations, bias and limits of agreement. [file 13054_2014_679_MOESM1_ESM.pdf]

Additional File 1

Patient	C_{rs}.CMV	C_{rs}(low-flow)	C_{rs}(low-flow)$_{desc}$	C_{rs}(PEEP Wave)
1	43	51	52	53
2	37	41	45	56
3	12	17	17	28
4	13	24	24	44
5	54	74	63	72
6	39	48	49	53
7	57	88	56	71
8	36	39	39	60
9	42	51	51	54
10	74	91	92	85
11	50	52	65	43
12	41	35	56	57
13	54	63	66	88
14	34	40	48	32
15	20	29	29	38
16	58	64	67	50
17	52	94	72	106
18	54	54	71	82
**mean**	**43**	**53**	**53**	**60**
**SD**	**16**	**22**	**18**	**20**

Table S1: Comparison of global respiratory system compliance (C_{rs}) obtained with the different reference methods and the PEEP wave maneuver. C_{rs}(CMV): C_{rs} calculated by dividing expiratory tidal volume by the difference between plateau airway pressure and PEEP during controlled mechanical ventilation (CMV). C_{rs}(low-flow): quasi-static C_{rs} obtained from the inspiratory part of the low-flow loop during CMV. C_{rs}(low-flow)$_{desc}$: quasi-static C_{rs} obtained from the corresponding expiratory part of the low-flow loop. All values are in ml/cmH_2O.

Patient	C_{rs}(low-flow)$_{ventral}$	C_{rs}(low-flow)$_{dorsal}$	C_{rs}(low-flow) per pixel	C_{rs}(low-flow) per pixel ventral	C_{rs}(low-flow) per pixel dorsal	C_{rs}(PEEP wave)$_{ventral}$	C_{rs}(PEEP wave)$_{dorsal}$	C_{rs}(PEEP wave) per pixel	C_{rs}(PEEP wave) per pixel ventral	C_{rs}(PEEP wave) per pixel dorsal
1	24	27	0.19	0.16	0.22	39	16	0.19	0.26	0.13
2	24	17	0.16	0.20	0.11	33	21	0.21	0.27	0.15
3	7	10	0.08	0.08	0.08	12	16	0.13	0.13	0.12
4	15	9	0.09	0.11	0.07	21	23	0.17	0.15	0.19
5	42	32	0.25	0.34	0.21	39	31	0.26	0.32	0.20
6	28	20	0.15	0.17	0.12	31	24	0.17	0.20	0.15
7	61	27	0.26	0.41	0.21	43	28	0.26	0.29	0.22
8	28	11	0.14	0.17	0.09	37	21	0.21	0.23	0.17
9	39	12	0.14	0.18	0.07	32	22	0.14	0.15	0.13
10	67	24	0.25	0.34	0.14	66	18	0.23	0.34	0.11
11	38	14	0.14	0.16	0.08	33	10	0.11	0.14	0.06
12	32	3	0.14	0.15	0.03	49	9	0.17	0.23	0.08
13	42	22	0.35	0.49	0.21	61	27	0.47	0.71	0.27
14	28	12	0.11	0.12	0.08	29	2	0.08	0.12	0.02
15	23	6	0.08	0.13	0.04	31	8	0.11	0.17	0.05
16	54	9	0.23	0.35	0.07	35	14	0.18	0.22	0.12
17	62	32	0.22	0.32	0.18	79	31	0.29	0.42	0.17
18	39	15	0.18	0.21	0.10	52	30	0.24	0.28	0.20
**mean**	**36**	**17**	**0.18**	**0.23**	**0.12**	**40**	**19**	**0.20**	**0.26**	**0.14**
**SD**	**16**	**9**	**0.07**	**0.11**	**0.06**	**16**	**8**	**0.09**	**0.13**	**0.06**

Table S2: Comparison of regional respiratory system compliance (C_{rs}). obtained with the low flow loop and the PEEP wave maneuver. All values are in ml/cmH_2O.

Comparison	r	p value for correlation	bias (mean of differences)	p value for mean of differences	95% limits of agreement
C_{rs}(PEEP wave) vs C_{rs}(low-flow)	0.80	< 0.0001	+6.8	0.06	-20 to +34
C_{rs}(PEEP wave) vs C_{rs}(CMV)	0.71	0.001	+17.1	0.0002	-13 to +47
C_{rs}(PEEP wave)$_{ventral}$ vs C_{rs}(low-flow)$_{ventral}$	0.77	0.0002	+4.8	0.08	-17 to +26
C_{rs}(PEEP wave)$_{dorsal}$ vs C_{rs}(low-flow)$_{dorsal}$	0.65	0.003	+2.1	0.24	-12 to +17
C_{rs}(PEEP wave) per pixel vs C_{rs}(low-flow) per pixel	0.86	< 0.0001	+0.03	0.03	-0.06 to +0.11
C_{rs}(PEEP wave)$_{ventral}$ per pixel vs C_{rs}(low-flow)$_{ventral}$ per pixel	0.81	< 0.0001	+0.03	0.09	-0.12 to + 0.19
C_{rs}(PEEP wave)$_{dorsal}$ per pixel vs C_{rs}(low-flow)$_{dorsal}$ per pixel	0.66	0.0027	+0.02	0.17	-0.09 to +0.12

Table S3: Correlation coefficients (r), p values for correlation, bias, p values for means of differences and 95% limits of agreement for the different comparisons between methods for determination of respiratory system compliance (C_{rs}) described in this paper. C_{rs}(PEEP wave): C_{rs}, determined with the PEEP wave maneuver. C_{rs}(CMV): C_{rs}, determined during volume-controlled mechanical ventilation as expiratory tidal volume divided by the difference between plateau airway pressure and PEEP. C_{rs}(low-flow): C_{rs}, determined with the low-flow maneuver.
